# No Effect of Body Size on the Frequency of Calling and Courtship Song in the Two-Spotted Cricket, *Gryllus bimaculatus*

**DOI:** 10.1371/journal.pone.0146999

**Published:** 2016-01-19

**Authors:** Atsushi Miyashita, Hayato Kizaki, Kazuhisa Sekimizu, Chikara Kaito

**Affiliations:** Laboratory of Microbiology, Graduate School of Pharmaceutical Sciences, The University of Tokyo, 3–1, 7-chome, Hongo, Bunkyo-ku, Tokyo, 113–0033, Japan; Virginia Commonwealth University, UNITED STATES

## Abstract

The relationship between body size and vocalization parameters has been studied in many animal species. In insect species, however, the effect of body size on song frequency has remained unclear. Here we analyzed the effect of body size on the frequency spectra of mating songs produced by the two-spotted cricket, *Gryllus bimaculatus*. We recorded the calling songs and courtship songs of male crickets of different body sizes. The calling songs contained a frequency component that peaked at 5.7 kHz. On the other hand, courtship songs contained two frequency components that peaked at 5.8 and 14.7 kHz. The dominant frequency of each component in both the calling and courtship songs was constant regardless of body size. The size of the harp and mirror regions in the cricket forewings, which are the acoustic sources of the songs, correlated positively with body size. These findings suggest that the frequency contents of both the calling and courtship songs of the cricket are unaffected by whole body, harp, or mirror size.

## Introduction

Acoustic signals are important communication tools for animals. Conspecific communications using acoustic signals are widely observed in both vertebrate and invertebrate species. In vertebrates, the auditory organ comprises several parts, including the tympanic membrane, the cochlea, and the auditory neurons that transduce the signals to the brain. In the vertebrate auditory system, the cochlea specifically recognizes the frequency content of incoming sound waves. Although the invertebrate auditory organ is evolutionarily different, its function to sense airwaves is surprisingly similar to that in vertebrates. Whether invertebrate auditory systems have a frequency-analyzing function is unclear, however, because supporting evidence is limited. The rainforest katydid was recently reported to analyze frequencies as well as the vertebrate cochlea [1], raising the possibility that the frequency content of invertebrate sounds contains biologically significant information, similar to that in vertebrates.

There are several proposals for the biologic meaning of sound pitch (frequency content) in animal acoustic signals. Vocalizations of primates, including humans, exhibit sexual dimorphism in their frequency content, which transmits sex information to conspecific individuals as well as other information regarding body size or body conditions [2]. In macaque or dog, the frequency content of the vocalization correlates with body size [3] [4]. In bat, the echolocation peak frequency correlates with male body mass, which is advantageous for mating [5]. Also, in a frog species, the dominant frequency of the call reflects the body size [6]. In contrast, there is no clear correlation between body size and frequency content in the songbird (fairy-wren, *Malurus coronatus coronatus*), although the lower limit of the frequency distribution may be associated with body size [7]. Similarly, in two sparrow species, *Junco hyemalis* and *Serinus serinus*, body size does not significantly correlate with the frequency content of their vocalizations [8]. Hence, whereas vocalizations of primates can transmit information about the speaker’s body size, it has remained unclear whether vocalizations of birds and more primitive species transmit size information *via* frequency components in their acoustic signals.

In this context, there is little evidence to support the notion that sounds produced by invertebrate species provide body size information. Crickets stridulate their forewings against each other to produce songs, and the vibration in the specific wing area produces compressional airwaves that propagate through the atmosphere, which conspecifics detect with their auditory systems. In *Acheta domesticus* [9], *Gryllus campestris* [10], and *Gryllus bimaculatus* [11], the frequency content of male calling songs does not significantly reflect body size. Also, another study demonstrated that the frequency content of the calling songs in three *Gryllus* species is unlikely to be affected by body size [12]. In the two-spotted cricket *G*. *bimaculatus*, males produce calling songs that attract distant females, and then courtship songs that induce nearby females to copulate. The courtship songs of *G*. *bimaculatus* contain high frequency components (15~20kHz) and a higher sampling rate (>40kHz) is required to record the song. Few reports have analyzed the effect of body size on the frequency pattern of the courtship song, as the pattern is more complicated than that of the calling song, and recording it requires a higher sampling rate. In the present study, we analyzed the courtship and calling songs of *G*. *bimaculatus* to evaluate the effect of body size on the frequency content of their vocalizations.

## Materials and Methods

### Crickets

The two-spotted cricket *G*. *bimaculatus* was purchased from Hachurui-club (Tokyo, Japan) or Tsukiyono-farm (Gunma, Japan). The crickets were maintained in plastic cages at 28°C. Commercial diet (‘Koorogi-food’, Tsukiyono Farm, Gunma, Japan) and water were supplied in the cage.

### Recorder and computer

A tablet PC (Nexus 7, Asustek computer, Taipei, Taiwan) was used to record the songs. A Macbook Pro (2.5 GHz Intel Core i5, OS X ver. 10.9, Apple Inc.) running R ver. 2.15.3 (https://www.r-project.org), or iMac (2.9 GHz Intel Core i5, OS X ver. 10.10, Apple Inc.) running R ver. 3.2.1 was used for data analysis. For sound analysis, we used the package “Seewave” [13], which works in both versions of R.

### Frequency response of the microphone

We analyzed the frequency response of the microphone used in this study (Nexus 7, Asustek computer, Taipei, Taiwan). A synthesized white noise was played through a speaker (FE166En, FOSTEX, Tokyo, Japan) and recorded by the microphone in an anechoic chamber. We confirmed that the generated white noise exhibited a flat spectrum around 1–24 kHz by a wide-range microphone (4191-C-001, Brüel & Kjær, Nærum, Denmark). The microphone was placed directly in front of the speaker (distance 50 cm) for each recording. After obtaining 30-s recordings of white noise and dark noise (no-sound background), the sound spectrum of the recorded sound files was analyzed (wave file). The response curve indicates that the tablet microphone we used in this study is capable of collecting sound signals from 0.5 kHz to 20 kHz (S1 Fig).

### Song recording and body size measurement

Five females and one male (day 7–14 adults [days were counted from their final molting into adult stage] were used for all recordings) were placed in a plastic cage. The song of each male cricket was recorded (sampling rate = 48,000Hz, 16 bit, wav file). Then, 2.00 s of calling and courtship song was extracted from the recorded file for each male using the free software Audacity (http://audacityteam.org). The cricket body mass was measured immediately before each recording using an analytical balance (AB54-S, Mettler-Toledo, Tokyo, Japan). The recordings were performed in a quiet room with fluorescent lights on 19–22 August 2013.

### Drawing spectrograms

Spectrograms of the cricket songs were plotted using the “spectro” function of package “Seewave” running on R. An oscillogram (a time wave) for each sound was also plotted below the spectrogram to clarify which part of the song corresponded to each frequency signal. The oscillogram was plotted using an option in the “spectro” function.

### Distribution of frequency components in the cricket song

The frequency distribution was analyzed on an R running package, “Seewave”. We loaded the 2-s wave files using the “readWave” function, applied a band-pass frequency filter (from 0.5 to 20 kHz) using the “ffilter” function, and calculated the frequency spectrum using the “spec” function. The amplitude values in each spectrum were normalized by its maximum as a default setting in the “spec” function. Parameters of calculation were as follows: window type = ‘hanning’, window length = 512. The calculations were performed for all individual songs, and the results were pooled and accumulated. A histogram was drawn from the pooled data, and the distribution curve was determined using the kernel density estimation with the “density” function in R.

### Correlation between the mean dominant frequency of each band and body size

The dominant frequency for each frequency band of the calling song or courtship song was calculated for each 2-s song wave as a set, applying a band-pass filter (4.0–9.0 kHz for calling, and 4.0–9.0 or 13.0–17.0 kHz for courtship). The “dfreq” function of the “Seewave” package in R was applied for this calculation (amplitude threshold = 10%). The relationship between the dominant frequency and body size were plotted (n = 50 for calling, and n = 53 for courtship). The effect of body size on song frequency was fit to a linear model followed by analysis of variance (ANOVA). The statistical information is summarized in Table 1.

**Table 1:** Evaluation of the body-size effect on frequency parameters of calling and courtship songs.

		Calling	Courtship	
	Band [kHz]	4.0–9.0	4.0–9.0	13.0–17.0
Parameters	Dominant frequency (Mean±SD) [kHz]	5.82±0.19	6.44±0.45	15.04±0.42
	Body mass (Mean±SD) [mg]	695±104	692±102	
	Number of crickets	50	53	
ANOVA	F-statistics	0.3237	1.405	4.343
	Degrees of freedom (Df)	1 and 48	1 and 51	1 and 51
	P-value	0.572	0.241	0.042
	Significance level	0.017	0.017	0.017
	Body-size effect	(-)	(-)	(-)

We recorded calling songs and courtship songs of *G*. *bimaculatus*, and calculated the dominant frequencies as described in the Materials and Methods. The effect of body size on the frequency parameters was evaluated using ANOVA. Degrees of freedom (Df), F-statistics, and p-values are shown in the table. The significance level was adjusted by Bonferroni correction.

### Measurement of harp and mirror areas in the male forewings

To measure the areas of the harp or mirror regions of the male forewings, we dissected the wings and scanned their images using CanoScan LiDE 210 (Canon Inc., Tokyo, Japan). The incorporated image files were loaded in ImageJ (National Institutes of Health, USA) and the areas were measured using the “Measure” command.

## Results

**Fig 1:**
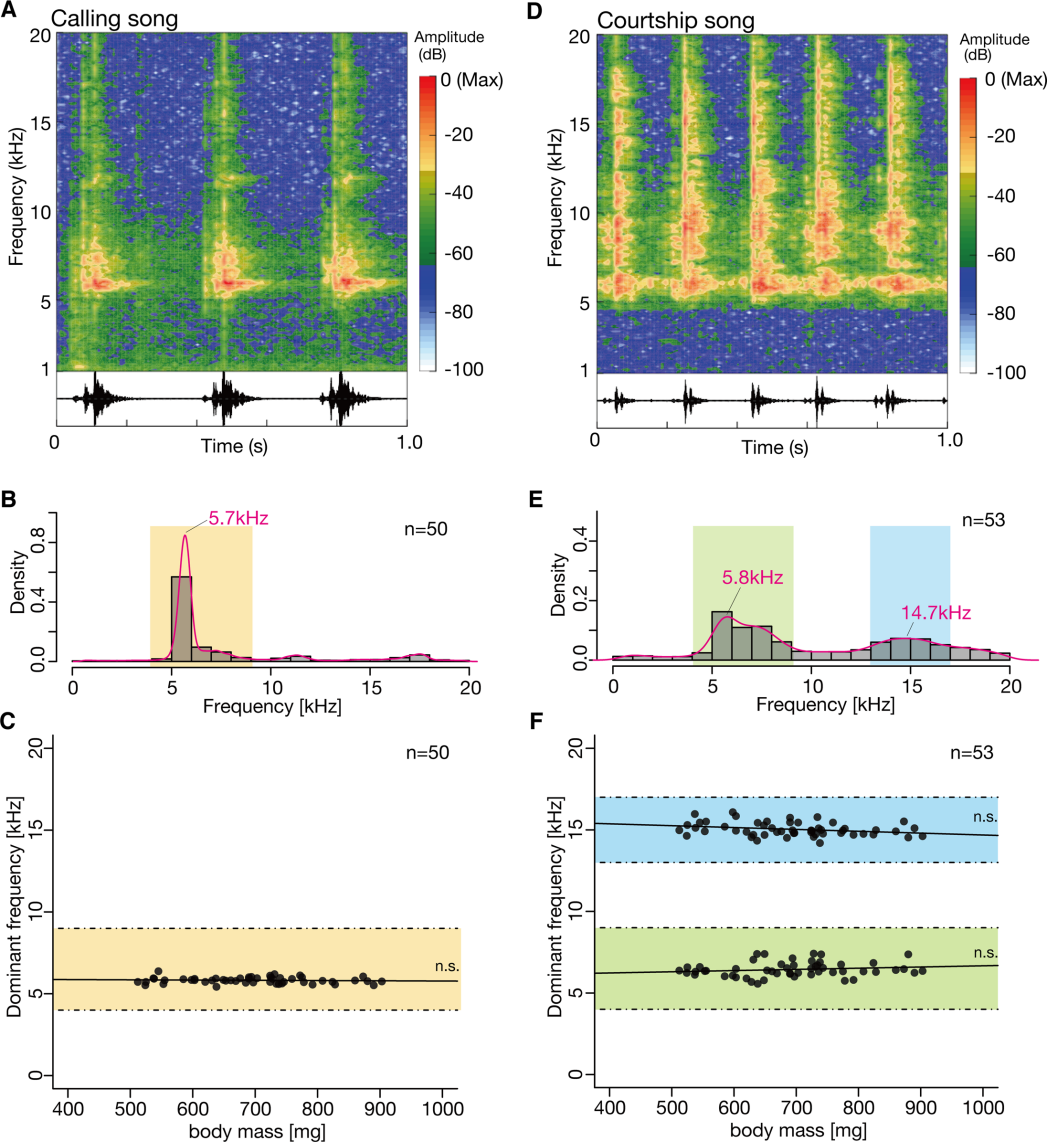
Analysis of the frequency spectra in calling and courtship songs, and body-size effects in *G*. *bimaculatus*. ***(A)*** *Upper panel*. The spectrogram of the calling song from a representative male. The vertical axis shows the frequency in kHz, and the horizontal axis shows the time in seconds. The color indicates the energy of each frequency component in dB (dBFS, decibels relative to full scale). The color scale is shown in the right panel. *Lower panel*. An oscillogram of the calling song from the representative male corresponding to the above spectrogram. The vertical axis shows the sound amplitude, and the horizontal axis shows the time [s]. (B) Distribution of the frequency component of calling songs (n = 50). The vertical axis shows the density, and the horizontal axis shows the frequency (kHz). The histogram (gray) and distribution curve from the kernel density estimate (magenta). The distribution curve shows a single peak at 5.7 kHz. The frequency band analyzed in the following experiment (Fig 1C) is indicated in orange. (C) Evaluation of the body-size effect on the dominant frequency of the calling song. The dominant frequency of the 2-s calling song was calculated for each male (n = 50) and plotted against the individual body mass. The vertical axis shows the dominant frequency (kHz), and the horizontal axis shows the body mass (mg). The background color of the band corresponds to the orange band shown in Fig 1B. No significant effect was detected (p = 0.572). Statistical information is summarized in Table 1. The significance level was adjusted by Bonferroni correction. (D) *Upper panel*. The spectrogram of the courtship song from a representative male. The vertical axis shows the frequency in kHz, and the horizontal axis shows the time in seconds. The color indicates the energy of each frequency component in dB (dBFS, decibels relative to full scale). The color scale is shown on the right panel. *Lower panel*. An oscillogram of the courtship song from the representative male corresponding to the above spectrogram. The vertical axis shows the sound amplitude, and the horizontal axis shows the time [s]. (E) Distribution of the frequency component of courtship songs (n = 53). The vertical axis shows the density, and the horizontal axis shows the frequency (kHz). The histogram (gray) and distribution curve from the kernel density estimate (magenta). The distribution curve shows two peaks at 5.8 and 14.7 kHz. The frequency bands analyzed in the following experiment (Fig 1C) are indicated in green or in blue. (F) Evaluation of the body-size effect on the dominant frequency of the courtship song. The dominant frequency of the 2-s courtship song was calculated for each male (n = 53) and plotted against the individual body mass. The vertical axis shows the dominant frequency (kHz), and the horizontal axis shows the body mass (mg). Each background color of the band corresponds to the green or blue band shown in Fig 1E. No significant effect was detected (p = 0.241 for lower band, p = 0.042 for higher band). Statistical information is summarized in Table 1. The significance level was adjusted by Bonferroni correction.

We recorded the calling songs of male crickets (2 s/male), and analyzed their frequency patterns. The spectrogram of each calling song exhibited a pattern in which high-energy spots around ca. 6 kHz, synchronizing with each pulses of the time wave were observed (Fig 1A). The frequency distribution of sounds pooled from 50 males exhibited a single peak at 5.7 kHz (Fig 1B). We then examined whether the dominant frequency of the peak content correlated with body size by analyzing the correlation between the cricket mass and the dominant frequency. The dominant frequency of individual crickets was constant, and did not significantly correlate (F(1,48) = 0.324 and p = 0.572) with cricket mass (Fig 1C and Table 1). These findings suggest that the frequency content in the calling song of *G*. *bimaculatus* does not reflect body size.

To investigate the frequency spectra of the courtship songs and their relationship with body size, we recorded courtship songs (2 s/male) and analyzed the frequency patterns. Compared with the spectrogram of the calling song, the spectrogram of each courtship song exhibited a relatively broader energy distribution for every courtship song pulse (Fig 1D). The frequency distribution of the songs from 53 cricket males showed two peaks, at 5.8 and 14.7 kHz (Fig 1E). We then examined whether the dominant frequency of the two components correlated with individual body size by applying band-pass filters and analyzing the correlation between cricket mass and the dominant frequency for each frequency band. The dominant frequency in each band of individual crickets was constant, and showed no significant correlation (F(1,51) = 1.045 and p = 0.241 for the lower band, (F(1,51) = 4.343 and p = 0.0422 for the higher band) with cricket mass (Fig 1F and Table 1). To eliminate the effects of multiple tests, we applied Bonferroni’s correction for multiple comparisons to the significance levels of statistical hypothesis tests. Our findings suggest that the frequency content in the courtship song of *G*. *bimaculatus* does not reflect body size.

**Fig 2:**
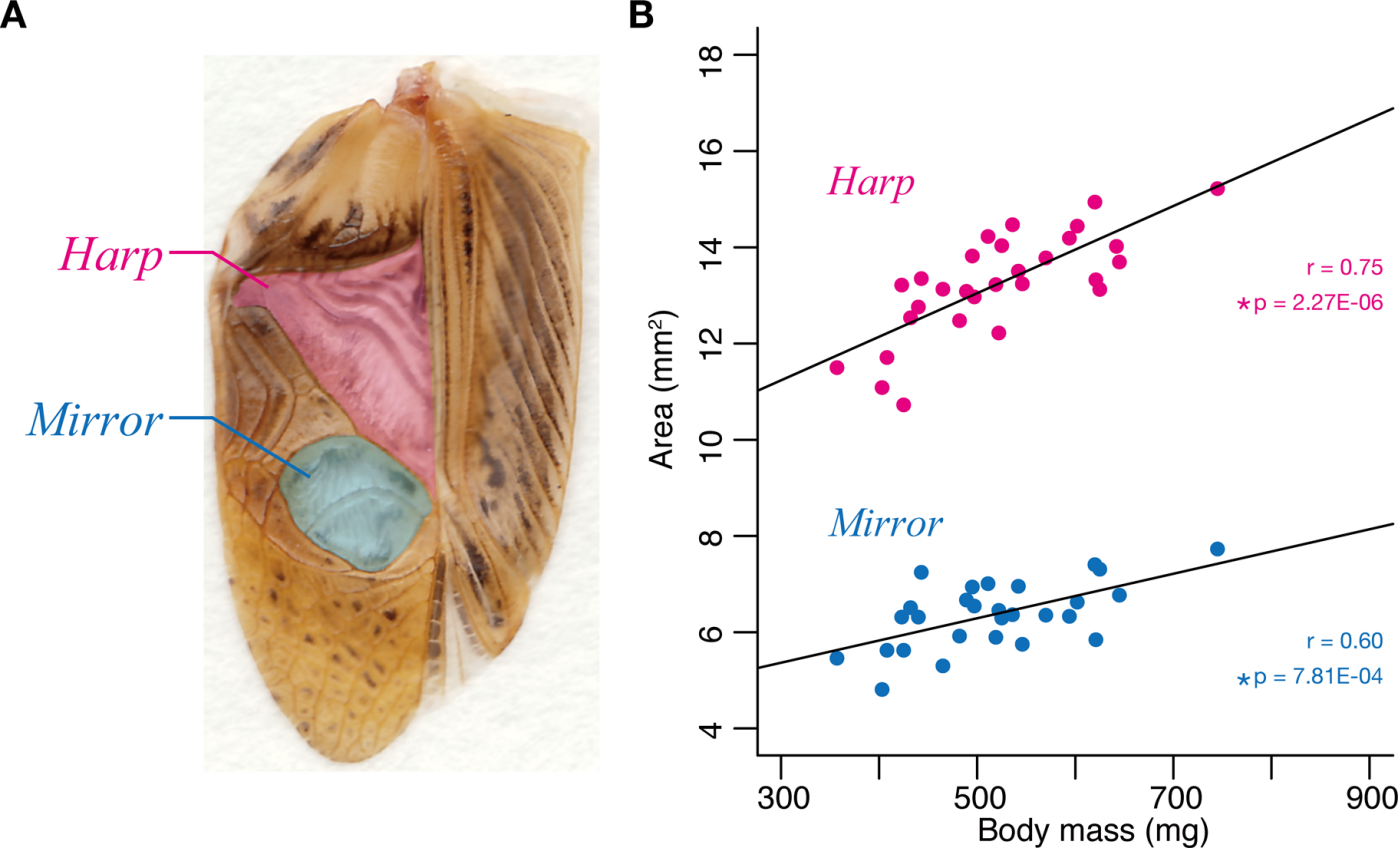
Relationship between forewing mirror size and whole body size. **(A)** Male forewings were dissected as shown in the panel. Measurements of the area (mm^2^) of the harp and mirror regions were obtained as indicated in magenta and blue, respectively. (B) Correlations between the harp size (magenta circle) or mirror size (blue circle) and whole body mass. The vertical axis shows the area (mm^2^) and the horizontal axis shows the cricket mass (mg). Both areas correlated significantly with the body mass. The correlation coefficient (r) and p values are presented in the graph.

We next examined whether the size of the sound sources correlate with body size in the cricket. We measured the areas of the harp and mirror regions in the cricket forewings (Fig 2A), which vibrate as males stridulate their forewings and are the sound source of the cricket song [14]. Body mass was significantly positively correlated with the harp area (r = 0.75, F(1,27) = 35.69, p = 2.27E-06, n = 29) and mirror area (r = 0.60, F(1,26) = 14.46, p = 7.81E-04, n = 28) of the male cricket forewings (Fig 2B). This finding suggests that the size of the sound source is proportional to cricket body size.

## Discussion

The present study revealed that the calling song of *G*. *bimaculatus* contains a pure frequency component at 5.7 kHz. Due to its pureness, the frequency pattern of the calling song was simple enough to analyze based on a single frequency component. On the other hand, the frequency pattern of the courtship songs was more complicated than that of the calling songs, making it difficult to analyze the frequencies with a single component. In this study, we applied the ordinary method in bioacoustics to the cricket song analyses, combining the pooled songs from a large number of crickets. Though the courtship song exhibited complicated frequency spectra, we demonstrated its average frequency distribution in an analysis of 53 male courtship songs. Further studies are needed to analyze each frequency component in cricket communication and its biologic meaning.

It is important to note that several studies of the calling song of *G*. *bimaculatus* in European countries have shown that the major frequency of *G*. *bimaculatus* calling songs is lower than 5.7 kHz (4.4–5.5 kHz[15], 5kHz [16], or 4.7 kHz [17, 18]). Another study performed in Japan, in contrast, reported that the major frequency of the calling song was 5.8 kHz [19]. At present, the reason(s) for the variation in the calling frequency is unclear. It may due to genetic variations in *G*. *bimaculatus* in different geographic areas or environmental adaptation to different areas as *G*. *bimaculatus* regulates the frequency components of their calling songs based on auditory feedback [20].

The present study demonstrated that the frequency content of the calling and courtship songs of the two-spotted cricket are robustly regulated, and are unaffected by body size. With regard to the calling songs, the findings of this study are consistent with those of previous studies reporting no significant effect of body size on cricket calling songs in *G*. *bimaculatus* [11]. Our findings also demonstrated that the sizes of the harp and mirror, which are the sound sources in the forewings, scale with the whole body size and are unlikely to be the frequency determinant in *G*. *bimaculatus*. A significant effect of harp size on the dominant frequency of calling songs in *G*. *campestris*, however, was suggested in previous studies [18, 21, 22], which raises the possibility that frequency-determining characteristics differ among *Gryllus* species. In crickets, the air vibrations generated by the stridulated forewings resonate somewhere in the body to produce the final sound. We speculate that a particular mechanism in this process functions to keep the frequency content stable, being unaffected by the cricket’s body size. Further elucidation of the physical acoustic mechanism from those points of view is important.

The efficacy of the female ear system for sound recognition is tuned for frequency components that are most abundant in the male songs [23]. The abundant frequency components induce female mating behavior by playback from a speaker [24]. Hence, the robustness of the frequency content in male songs is associated with mating efficacy, suggesting that females would likely ignore a male who produces a song that differs from the average. Male size is thought to be the key parameter in female preference in crickets [10], indicating that the signals that transmit size information are coded in a manner other than the mean frequency of the calling or courtship songs. Other song parameters, such as sound intensity, may transmit size information. Further studies are needed to reveal how cricket females recognize male characteristics, including body size, based on song patterns other than the frequency content analyzed in this study.

## Supporting Information

S1 Fig The frequency response of the microphone used in this study. The frequency response of the microphone used in this study was determined. The frequency distributions of sound data of white noise (blue) and dark noise (orange) were analyzed. The horizontal axis shows frequency in Hz, and the vertical axis shows the signal level. Both axes are log-scaled.
(TIF)
