# Supplementary figures and images for: No Effect of Body Size on the Frequency of Calling and Courtship Song in the Two-Spotted Cricket, Gryllus bimaculatus
Source: PLoS One. 2016 Jan 19;11(1):e0146999. doi: 10.1371/journal.pone.0146999 (PMC4718538; doi:10.1371/journal.pone.0146999)

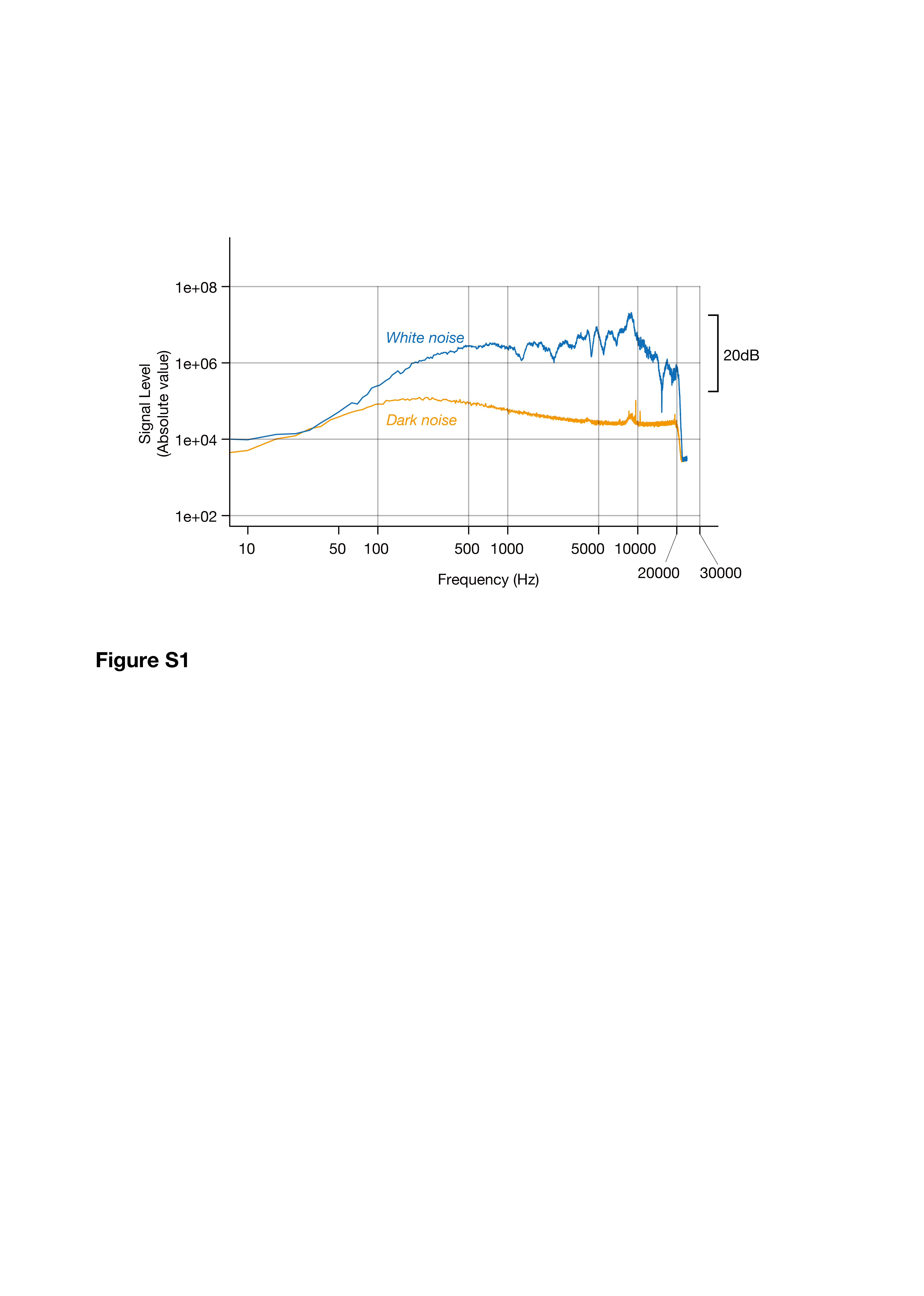

Supplement: S1 Fig — The frequency response of the microphone used in this study was determined. The frequency distributions of sound data of white noise (blue) and dark noise (orange) were analyzed. The horizontal axis shows frequency in Hz, and the vertical axis shows the signal level. Both axes are log-scaled. (TIF) [file pone.0146999.s001.tif]
